# DOCK2 and phosphoinositide-3 kinase δ mediate two complementary signaling pathways for CXCR5-dependent B cell migration

**DOI:** 10.3389/fimmu.2022.982383

**Published:** 2022-10-19

**Authors:** Stefanie Wissmann, Bettina Stolp, Ana Marcos Jímenez, Jens V. Stein

**Affiliations:** ^1^ Department of Oncology, Microbiology and Immunology, University of Fribourg, Fribourg, Switzerland; ^2^ Department for Infectious Diseases, Integrative Virology, Center for Integrative Infectious Disease Research, University Hospital Heidelberg, Heidelberg, Germany; ^3^ Department of Immunology, Biomedical Research Institute La Princesa Hospital, Madrid, Spain

**Keywords:** B cell migration, CXCR5 (C-X-C motif chemokine receptor 5), intravital 2-photon microscopy, phosphoinoside-3-kinase, DOCK2

## Abstract

Naive B cells use the chemokine receptor CXCR5 to enter B cell follicles, where they scan CXCL13-expressing ICAM-1^+^ VCAM-1^+^ follicular dendritic cells (FDCs) for the presence of antigen. CXCL13-CXCR5-mediated motility is mainly driven by the Rac guanine exchange factor DOCK2, which contains a binding domain for phosphoinositide-3,4,5-triphosphate (PIP3) and other phospholipids. While p110δ, the catalytic subunit of the class IA phosphoinositide-3-kinase (PI3K) δ, contributes to CXCR5-mediated B cell migration, the precise interdependency of DOCK2, p110δ, or other PI3K family members during this process remains incompletely understood. Here, we combined *in vitro* chemotaxis assays and *in vivo* imaging to examine the contribution of these two factors during murine naïve B cell migration to CXCL13. Our data confirm that p110δ is the main catalytic subunit mediating PI3K-dependent migration downstream CXCR5, whereas it does not contribute to chemotaxis triggered by CXCR4 or CCR7, two other chemokine receptors expressed on naïve B cells. The contribution of p110δ activity to CXCR5-driven migration was complementary to that of DOCK2, and pharmacological or genetic interference with both pathways completely abrogated B cell chemotaxis to CXCL13. Intravital microscopy of control and gene-deficient B cells migrating on FDCs confirmed that lack of DOCK2 caused a profound migration defect, whereas p110δ contributed to cell speed and directionality. B cells lacking active p110δ also displayed defective adhesion to ICAM-1; yet, their migration impairment was maintained on ICAM-1-deficient FDCs. In sum, our data uncover two complementary signaling pathways mediated by DOCK2 and p110δ, which enable CXCR5-driven naïve B cell examination of FDCs.

## Introduction

Naïve follicular B cells are highly motile cells, which scan ICAM-1^+^ VCAM-1^+^ follicular dendritic cells (FDCs) for the presence of microbial antigen and the initiation of humoral responses. The chemokine receptor CXCR5 is critical for naïve B cell access to follicles, where FDCs, together with other stromal cells such as marginal reticular cells, produce its only ligand CXCL13 (1, 2). Furthermore, CXCR5 promotes together with the ICAM-1 receptor LFA-1 dynamic B cell surveillance of FDCs (3, 4). The lymphocyte-expressed guanine exchange factor (GEF) DOCK2 is a key signaling molecule for Rac activation and F-actin polymerization downstream of chemokine receptors in lymphocytes. In the absence of DOCK2, *in vitro* T and B cell migration towards homeostatic chemokines is strongly compromised, although residual migration persists (5). Accordingly, direct observation of peripheral lymph nodes (PLN) using intravital twophoton microscopy (2PM) uncovered that follicular accumulation and interstitial motility are substantially reduced but not completely abolished in DOCK2^-/-^ deficient B cells (6).

DOCK family proteins contain two DOCK homology regions (DHR), of which DHR1 is involved in phospholipid binding for membrane localization and DHR2 mediates the GEF activity (7–9). The DHR1 domain of DOCK2 binds the phosohoinositide-3-kinase (PI3K) product phosphoinositide-3,4,5-triphosphate (PIP3) as well as phosphatidic acid (PA). In B cells, the relation between DOCK2 and PI3K activity remains unclear to date. Whereas DOCK2 activity is not required for PI3K activation (5) and PI3K inhibition does not affect DOCK2-mediated migration in T cells (10), neutrophil-expressed DOCK2 regulates migration through PIP3-dependent membrane translocation and Rac activation (11). Along the same line, the class IA p110δ catalytic subunit is involved in B cell chemotaxis towards CXCL13 not but CCL19, CCL21 and CXCL12 (12), and regulatory subunits of class IA are required for basal B cell motility *in vivo* (13). A potential participation of class I catalytic subunits besides p110δ during CXCR5-mediated B cell chemotaxis has not been examined yet, despite evidence for activation of additional class I PI3K family members downstream of G-protein coupled receptors (14).

Here, we examined the migratory behavior of B cells carrying mutations in DOCK2 and the catalytic site of p110δ (p110δ^D910A/D910A^), in combination with PI3K-specific pharmacological inhibitors, to dissect their contribution for CXCL13-elicited motility. Among class I PI3K catalytic subunits, we confirm a key contribution of p110δ to CXCR5- but not CXCR4 and CCR7-dependent migration. DOCK2 and p110δ activity comprised two complementary pathways for CXCR5-triggered B cell migration, and inhibition of both factors completely abolished chemotaxis. We corroborated our *in vitro* findings using intravital imaging of interstitial B cell scanning of FDCs. Finally, we found that while LFA-1 activity is reduced in the absence of catalytically active p110δ, the interstitial migration defect of p110δ^D910A/D910A^ B cells is maintained on ICAM-1-deficient FDCs. In sum, our study sheds light on intracellular signaling pathways governing CXCR5-driven follicular B cell motility, a prerequisite for the unfolding of humoral immune responses.

## Results

### p110δ is the dominant class I PI3K mediating B cell chemotaxis to CXCL13

**Figure 1:**
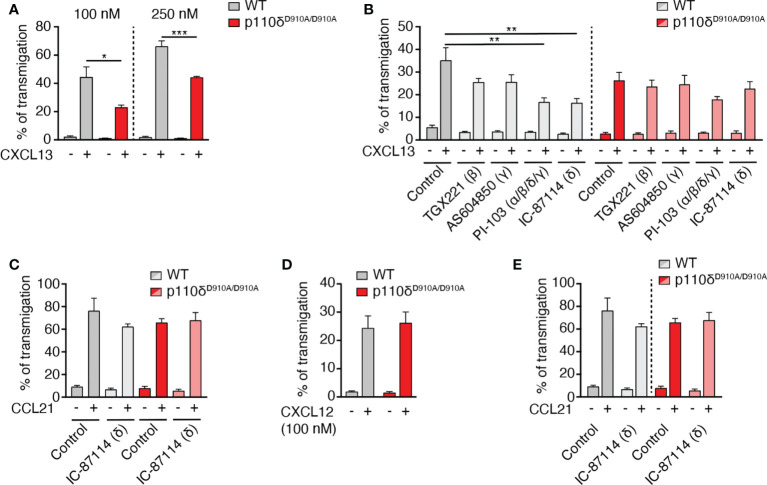
B cell migration to CXCL13 but not CCL21 or CXCL12 is mediated by p110δ activity. **(A)**. Chemotaxis of primary murine WT and p110δ^D910A/D910A^ B cells to 100 and 250 nM CXCL13 using Transwell assays. **(B)**. Chemotaxis of WT and p110δ^D910A/D910A^ B cells to 100 nM CXCL13 in presence of subunit-specific PI3K inhibitors. **(C)**. Chemotaxis of WT and p110δ^D910A/D910A^ B cells to 100 nM CCL21 in presence or absence of p110δ inhibitor. **(D)**. Chemotaxis of WT and p110δ^D910A/D910A^ B cells to 100 nM CXCL12. **(E)**. Chemotaxis of primary murine WT and p110δ^D910A/D910A^ T cells to 100 nM CCL21. Data in **(A–D)** are shown as mean ± SEM pooled from 3-5 independent experiments (**E**: 2 independent experiments) performed in duplicates and analyzed using unpaired t-test **(A, D)** or ANOVA with Dunnett’s test for “control + chemokine” conditions **(B, C, E)**. *p < 0.05; **p < 0.01; ***p < 0.001.

The class I PI3K family member p110δ contributes to directed B cell migration towards CXCL13 (12). Using Transwell assays, we confirmed a role for the catalytic activity of p110δ for *in vitro* chemotaxis of primary murine B cells towards CXCL13, which was particularly evident at lower chemokine concentrations (reduction of 48% at 100 nM and 33% at 250 nM CXCL13 for p110δ^D910A/D910A^ B cells as compared to WT B cells, respectively; **Figure 1A**). To address whether additional catalytic subunits might contribute to WT and p110δ^D910A/D910A^ B cell migration, we performed chemotaxis assays in presence of the p110β inhibitor TGX221, the p110γ inhibitor AS604850, the p110α/β/δ/γ inhibitor PI-103 and, as control, the p110δ inhibitor IC-87114. These data uncovered a decrease of WT B cell chemotaxis towards 100 nM CXCL13 only with PI-103 and IC-87114 (52% and 54% inhibition, respectively), while none of the inhibitors had a significant effect on p110δ^D910A/D910A^ B cell migration (**Figure 1B**). These findings suggest that other class IA and IB subunits do not substantially contribute to primary B cell migration towards CXCL13. The promigratory signaling function of p110δ was restricted to CXCR5, since B cell migration to CCR7 and CXCR4 ligands remained unchanged by genetic or pharmacological inhibition of its activity (**Figures 1C**, **D**), as reported (12). Similarly, CCR7-mediated primary T cell chemotaxis was not reduced by genetic or pharmacological inhibition of the catalytic activity of p110δ (**Figure 1E**).

### DOCK2 and p110δ comprise two complementary pathways for CXCR5-mediated B cell migration

**Figure 2:**
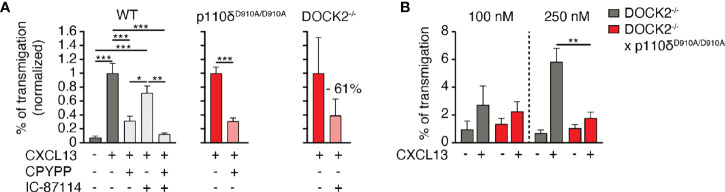
p110δ and DOCK2 comprise two complementary pathways for B cell migration to CXCL13. **(A)**. Normalized chemotaxis of WT, p110δ^D910A/D910A^ and DOCK2^-/-^ B cells to 100 nM CXCL13 in presence of DOCK2 and p110δ inhibitors using Transwell assays. **(B)**. Chemotaxis of DOCK2^-/-^ and DOCK2^-/-^ x p110δ^D910A/D910A^ B cells to 100 and 250 nM CXCL13. Data in A and B are shown as mean ± SEM pooled from 2-5 experiments performed in duplicates and analyzed using ANOVA with Tukey’s post-test **(A)** or an unpaired student’s t-test **(B)**. *p < 0.05; **p, < 0.01; ***p < 0.001.

We next examined the potential relationship of DOCK2 and p110δ during *in vitro* B cell chemotaxis towards CXCL13, given that DOCK2 contains a PIP3 binding domain. In a first set of experiments, we treated WT B cells separately or in combination with IC-87114 and CPYPP, which blocks the GEF activity of DOCK2 by binding to its catalytic DHR2 domain (15). These data showed that DOCK2 and p110δ comprised two complementary pathways for CXCR5-mediated chemotaxis, since only simultaneous treatment with both inhibitors completely abolished migration (**Figure 2A**). A blocking effect of CPYPP and IC-87114 was also observed for CXCL13-induced migration of p110δ^D910A/D910A^ and DOCK2^-/-^ B cells, respectively (**Figure 2A**).

Since inhibitors are often not entirely specific, we generated p110δ^D910A/D910A^ x DOCK2^-/-^ mice to corroborate our findings in a genetic model. Double-deficient mice were born at sub-mendelian ratios and showed growth retardation (not shown). Owing to the difficult breeding, we could isolate cells from these mice for only limited amounts of chemotaxis assays. In these experiments, residual migration of DOCK2^-/-^ B cells to 250 nM CXCL13 was abolished when p110δ activity was additionally compromised (**Figure 2B**). Taken together, these data suggest that DOCK2 and p110δ act in largely non-overlapping pathways downstream of CXCR5 signaling.

### p110δ activity contributes to B cells speed and directionality during follicular migration

**Figure 3:**
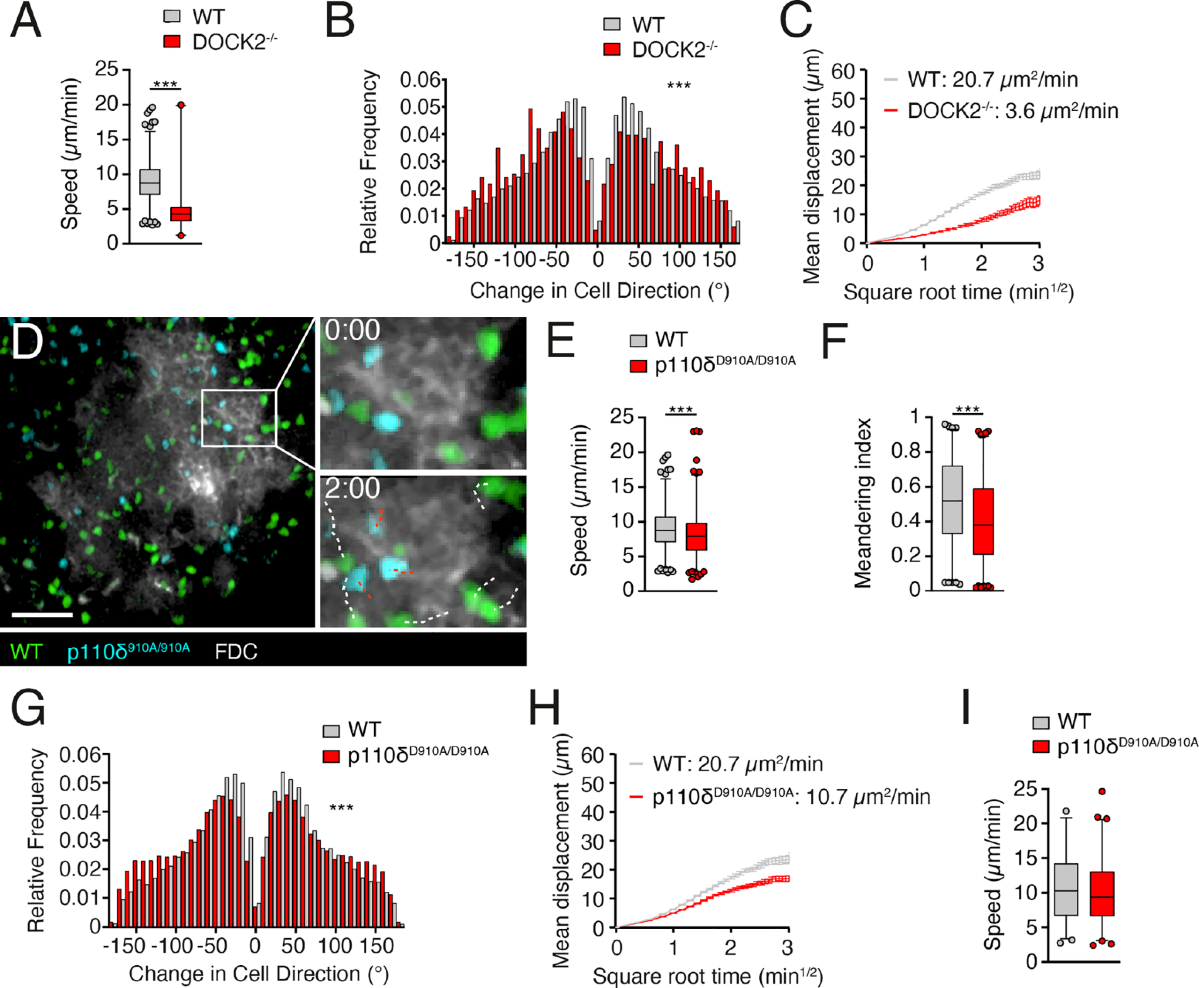
p110δ contributes to B cell scanning in follicles. **(A, B)**. Average track speeds and **(A)** and tuning angle distribution **(B)** of WT and DOCK2^-/-^ B cells. **(C)**. Mean displacement versus time of WT and DOCK2^-/-^ B cells. The motility coefficient (MC) is indicated. **(D)**. 2PM image from the FDC (white)-positive B cell follicle containing WT (green) and p110δ^D910A/D910A^ (blue) B cells after adoptive transfer in WT recipients. The boxed area is shown magnified for the 0 and 2 min time points, with WT B cell tracks shown in white and p110δ^D910A/D910A^ B cell tracks shown in red. Number of tracks analyzed: WT, n = 708; p110δ^D910A/D910A^, n = 835; DOCK2^-/-^, n = 104. Scale bar, 40 µm. **(E–G)**. Average track speeds **(E)**, meandering index **(F)** and tuning angle distribution **(G)** of WT and p110δ^D910A/D910A^ B cells. **(H)**. Mean displacement versus time of WT and p110δ^D910A/D910A^ B cells. **(I)**. Average track speeds of WT and p110δ^D910A/D910A^ T cells (n= 298 and 370 tracks, respectively) in intravital imaging of lymphoid tissue. Data from one **(A, B)**, two **(I)** or 5 **(E–H)** independent experiments (= recipient mice) and analyzed by an unpaired t-test **(A, E, I)** or Mann-Whitney test **(B, F, G)**. ***p < 0.001.

CXCR5 is required for B cell entry to B cell follicles (1), where it contributes to fast motility (4). This motility is in large part driven by DOCK2-mediated Rac activation, since DOCK2^-/-^ B cells show substantially reduced interstitial movement (6). Using 2PM of popliteal PLN containing adoptively transferred B cells (4), we confirmed a substantial drop in mean speeds in DOCK2-deficient B cells (from 7.9 ± 4.7 to 4.0 ± 2.9 µm/min for WT and DOCK2^-/-^ B cells, respectively; **Figure 3A**). This decline in speed was accompanied by broader turning angles and a low motility coefficient (MC), a proxy for a cell’s ability to scan an area (20.7 and 3.6 µm^2^/min for WT and DOCK2^-/-^ B cells, respectively; **Figures 3B**, **C**), in line with our previous observations (6).

We then examined whether p110δ contributed to B cell scanning of B cell follicles *in vivo*. In contrast to DOCK2^-/-^ B cells, WT and p110δ^D910A/D910A^ B cells accumulated efficiently in B cell follicles (**Figure 3D** **;** **Supplemental Movie 1**). However, p110δ^D910A/D910A^ B cells moved with decreased speeds and less directionality compared to WT B cells, as measured by meandering index and turning angle distribution (**Figures 3E-G**). As a result, p110δ^D910A/D910A^ B cells had an approximately 50% reduction of their MC compared to WT B cells (**Figure 3H**). In contrast, interstitial p110δ^D910A/D910A^ T cell migration speeds were similar to those of WT T cells (**Figure 3I**). These data support a contribution of p110δ activity to B cell motility along the FDC network inside B cell follicles.

### Reduced speed and directionality of p110δ^D910A/D910A^ B cells are maintained in the absence of stromal ICAM-1

**Figure 4:**
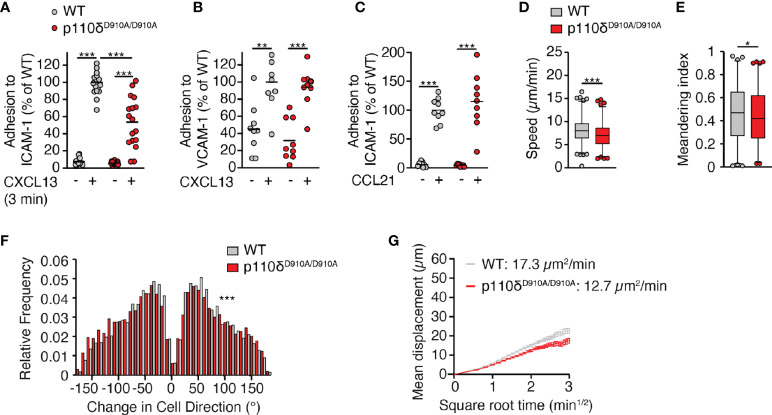
p110δ mediates interstitial B cell motility independent of ICAM-1. **(A, B).** Adhesion of WT and p110δ^D910A/D910A^ B cells to ICAM-1 **(A)** or VCAM-1 **(B)** measured 3 min after CXCL13 addition. **(C)**. Adhesion of WT and p110δ^D910A/D910A^ T cells to ICAM-1 measured 3 min after CCL21 addition. Values in A-C are normalized to the number of adhered cells in wells with chemokine addition. **(D–F)**. Average track speeds **(D)**, meandering index **(E)** and turning angle distribution **(F)** of WT and p110δ^D910A/D910A^ B cells in ICAM-1^-/-^ recipients. Number of tracks analyzed: WT, n = 490; p110δ^D910A/D910A^, n = 427. **(G)**. Mean displacement versus time of WT and p110δ^D910A/D910A^ B cells in ICAM-1^-/-^ recipients. The motility coefficient (MC) is indicated. Data in **(A–C)** are from 3-6 and in **(D-F)** from 2 independent experiments and analyzed using unpaired t-test with Welch correction **(A–C)**, unpaired t-test **(D)** and Mann-Whitney test **(E, F)**. *p < 0.05; **p < 0.01; ***p < 0.001.

In addition to CXCR5, LFA-1 contributes to B cell motility on ICAM-1^+^ VCAM-1^+^ FDCs, whereas α4 integrins play no detectable role (16). In line with this, β2 integrin-dependent *in vitro* leukocyte migration requires Syk-mediated p110δ translocation to the leading edge (17). Given the comparable impact of defective LFA-1 and p110δ activity on dynamic B cell motility parameters, we examined whether p110δ activity mediated its promigratory effect *via* LFA-1 activation. In support of this, an analysis of CXCL13-triggered *in vitro* adhesion to FDC-expressed adhesion molecules uncovered a reduction in p110δ^D910A/D910A^ B cell binding to ICAM-1 but not VCAM-1 (**Figures 4A**, **B**). Again, this adhesion defect was restricted to B cells, since p110δ^D910A/D910A^ T cell adhesion to ICAM-1 was not impaired (**Figure 4C**). We transferred WT and p110δ^D910A/D910A^ B cells into ICAM-1^-/-^ recipients, the main stromal LFA-1 ligand used by B cells in lymphoid tissue (16). We hypothesized that WT and p110δ^D910A/D910A^ B cells would show similar migration speeds if p110δ exerted its promigratory effect *via* LFA-1. However, we still observed reduced migration speeds, meandering index and increased turning angles in p110δ^D910A/D910A^ B cells compared to WT B cells (**Figures 4D–F**). As a consequence, their MC remained lower than the one of WT B cells (**Figure 4G**). These data suggest that the migration defect of p110δ^D910A/D910A^ B cells is largely independent of LFA-1-mediated adhesion to the FDC network. In sum, our data uncover a role for p110δ activity during B cell migration in lymphoid tissue, which is less pronounced than the effect caused by absence of DOCK2.

## Discussion

CXCR5-driven B cell chemotaxis to CXCL13 is critical for the development of humoral immune responses, as it enables efficient surveillance of FDCs and the proper formation of germinal centers (1, 18, 19). Here, we examined the intracellular wiring of CXCR5 that transmits biochemical input into a promigratory response. Our *in vitro* chemotaxis assays confirmed a critical role for the Rac GEF DOCK2 in mediating robust B cell chemotaxis to CXCL13, while p110δ participates in a complementary signaling module. These observations were recapitulated *in vivo*, suggesting the existence of two signaling pathways underlying CXCL13-mediated motility. The requirement of a PI3Kδ-dependent signaling module appears restricted to CXCR5, since migration to CXCR4 and CCR7 ligands was not impaired.

Intravital imaging has uncovered that B cell adhesion in PLN high endothelial venules (HEV) is more strongly attenuated by the absence of DOCK2 as compared to adhesion in Peyer’s patch (PP) HEV, although in both cases there is a significant reduction in B cell attachment (10). In contrast, lack of PI3Kδ activity mainly affects B cell homing to mesenteric lymph nodes (MLN) and PP, while these cells show normal homing to PLN (12). This may be due to the fact that CXCR5 plays a more prominent role for B cell homing to MLN and PP as compared to PLN, where CCR7 and CXCR4 play compensating roles (20, 21). Thus, the cooperative action of DOCK2 and PI3Kδ activity appears to extend to CXCR5-driven B cell entry into secondary lymphoid organs.

The parallel occurrence of a major, DOCK2-dependent pathway and a minor PI3K-dependent pathway in B cells mirrors observations made in naïve T cells. In T cells, the class IB p110γ isoform mediates DOCK2-independent migration *via* a pathway involving the PIP3-binding pleckstrin homology (PH)-domain containing Tec family kinase Itk (6, 10, 12, 22). Accordingly, DOCK2^-/-^ x p110γ^-/-^ T cells show no residual migration to CCL21 (10). In combination with the lack of p110δ involvement during naïve T cell migration *in vitro* and *in vivo*, our data support a model where p110γ and p110δ catalytic subunits contribute to T and B cell motility in a subset-specific manner. Of note, during CD4^+^ T cell differentiation to follicular helper T cells (T_FH_), p110δ signals downstream ICOSL induce T_FH_ precursor migration into the B cell follicles (23), suggesting context-specific roles for PI3K family members during lymphocyte positioning within lymphoid organs.

It remains incompletely understood how p110δ signaling contributes mechanistically to B cell migration downstream CXCR5, although Rac activation is likely to be required (24). A conceivable scenario is that PI3Kδ activates the B cell homologue of Itk, the PH-domain-containing Btk (25). In chronic lymphocytic leukemia (CLL) cell lines, pharmacological blockade of either p110δ or Btk reduces migration to CXCL13 (26, 27). Btk is linked to Vav phosphorylation, leading to downstream WASP activation and F-actin remodeling (28).

Unexpectedly, we found that the defect of p110δ^D910A/D910A^ B cell was maintained in lymphoid microenvironment lacking stromal ICAM-1, despite the known involvement of Syk-p110δ signaling during β2-integrin-mediated migration on 2D surfaces (17). A plausible explanation is that akin to naïve T cell migration within lymph node parenchyma, the main role for LFA-1 might be for generation of traction forces without inducing substantial adhesion (29). In the 3D confined environment of lymphoid tissue, substrate adhesion is externally enforced by juxtaposed cells, thus compensating for reduced LFA-1 activity.

The robust DOCK2-driven migration of p110δ^D910A/D910A^ B cells to CXCL13 raises the question whether PI3K-mediated signaling has additional roles beyond promoting cell motility. Another open point is whether PI3Kδ signaling might be involved in signal transduction downstream GPR183, although this receptor appears to have an inhibitory effect on CXCR5-mediated migration (4). In T cells, Itk contributes to homeostasis, suggesting a role for PI3K-dependent signaling in maintaining peripheral T cell numbers (22). Similarly, it is conceivable that CXCR5-mediated PI3K activation contributes to B cell homeostasis, in line with the well-documented role of this pathway for survival (30). In addition, the selective integration of p110δ signaling downstream CXCR5, but not other receptors for homeostatic chemokines, might facilitate B cell activation by feeding into the BCR-triggered PI3K-Btk signaling axis. A similar costimulatory signaling pathway was reported for CCL21 during T cell activation (31).

In sum, our data uncover dual signaling pathways mediating physiological CXCR5-triggered B cell motility that underpins rapid detection of cognate antigens presented on FDCs. Given that small tyrosine kinase inhibitors targeting p110δ and Btk are widely used in the treatment of leukemias (32–34), it is of clinical interest to understand potential implications on the patients’ immune system.

## Materials and methods

### Mice

Six to 12-week-old male and female DOCK2^-/-^ (5), p110δ^D910A/D910A^ (35), DOCK2^-/-^ x p110δ^D910A/D910A^ and ICAM-1^-/-^ (36) mice on the C57BL/6 background were bred at the University of Bern and Fribourg. Sex-and age-matched C57BL/6 mice (Harlan, The Netherlands) were used as WT lymphocyte donors or recipient mice. All experiments were performed in accordance with federal animal experimentation regulations and approved by the corresponding cantonal committee.

### Isolation and labeling of primary lymphocytes

B or T cells from PLN, MLN and spleen were purified by negative immunomagnetic cell sorting according to manufacturer’s instructions (Dynal or Stemcell technologies; purity of >95%). For intravital imaging experiments, purified B or T cells (5 x 10^6^) from C57BL/6 or genetically modified mice were fluorescently labeled for 15 min at 37°C with Cell Tracker blue (20 µM final concentration), Cell Tracker orange (5 µM), Cell Tracker green (3 µM) or CFSE (2.5 µM), washed and injected intravenously into sex-matched C57BL/6 recipient mice, together with 10-15 µg Alexa633-conjugated MECA-79 to label high endothelial venules. Dyes were switched between experiments to control for non-specific effects.

### Chemotaxis

CCL21 and CXCL12 were from Peprotech, and CXCL13 was purchased from R&D systems. Chemotaxis assays were carried out using Transwell chambers (5 µm pore size; CoStar) adding 100 µl cell suspension (5 x 10^6^ cells/ml) in complete medium (RPMI/10% FCS/standard supplements) to the top chamber and indicated amounts of chemokine in the bottom chamber. After 2 h at 37°C, 7% CO_2_, the percentage of migrated cells was calculated by flow cytometry after comparing with a precalibrated bead standard (Sigma-Aldrich) and correcting for variations in input concentrations. The DOCK2 inhibitor CPYPP (Selleck) was used at 40 µM throughout the chemotaxis assay (15). The isoform-specific PI3K inhibitors TGX221 (0.1 µM final conc.; Tocris), PI-103 (1 µM; Tocris), AS604850 (1 µM; Selleck),and IC-87114 (0.5 µM; Selleck) were present throughout the chemotaxis assay.

### Adhesion assay

Adhesion assays were performed as described (10). In brief, purified B or T cells were allowed to settle on 8-well-slides coated with 1.5 µg/ml murine ICAM-1 or 2.5 µg/ml VCAM-1 (R&D Systems). Chemokine was added at a final concentration of 1 µM for 3 min. Slides were rinsed with PBS to wash off unbound cells, fixed in glutaraldehyde, and the number of adherent cells was determined at the site of chemokine addition.

### Twophoton intravital microscopy

Fluorescently labeled WT and genetically modified B cells were adoptively transferred into WT or ICAM-1^-/-^ recipients 12-48 h before 2PM recording. In some experiments, PE-conjugated anti-CD35 mAb (0.5 µg in 10 µl PBS/mouse) was injected into the footpad 12 h before 2PM to label the FDC network of the draining popliteal PLN. Recipient mice were surgically prepared to expose the right popliteal PLN, which was kept at 36-38°C. Mice were then transferred to an Olympus BX50WI fluorescence microscope attached to a 2PM scanner (TrimScope system, LaVision Biotec, Bielefeld, Germany) equipped with an 20X objective (Olympus, NA 0.95). For four-dimensional analysis of cell migration, 8-16 z-stacks (spacing 4 µm) of 200-300 µm x-y sections were acquired every 20 s for 20 to 30 min, with typically 3-4 distinct areas recorded per preparation. Image sequences were transformed into volume-rendered four-dimensional movies using Volocity (Perkin Elmer) or Imaris (Bitplane), which was also used for semi-automated tracking of cell motility in three dimensions. From x, y and z coordinates of cell centroids, parameters of cellular motility were calculated as described previously. In brief, the track speed is depicted as average speed, with each dot representing one track. Owing to the large number of tracks, they are shown as box and whisker plots with whiskers covering 1-99% of data points. For turning angles and motility coefficients, we used MatLab scripts kindly provided by Dr. Sarah Henrickson and Prof. Ulrich H. von Andrian (Harvard University, Boston, USA). In some experiments, purified WT and p110δ^D910A/D910A^ T cells were transferred into WT recipients and their migratory behavior was analyzed in the T cell area as above.

### Statistical analysis

The student’s t-test or ANOVA were used to determine statistical significance (Prism, GraphPad). Statistical significance was set at p < 0.05.

## Data availability statement

The raw data supporting the conclusions of this article will be made available by the authors, without undue reservation.

## Ethics statement

The animal study was reviewed and approved by Canton of Fribourg and the Canton of Bern.

## Author contributions

SW, BS, and AM performed experiments. JS supervised the work and wrote the manuscript with input from all coauthors. All authors contributed to the article and approved the submitted version.

## Funding

This work was funded by Swiss National Foundation (SNF) project grants 31003A_172994, 310030_200406, Sinergia project grant CRSII5_170969, and the San Salvatore Foundation (to JVS), Leopoldina fellowship LPDS 2011-16 and the Deutsche Forschungsgemeinschaft project number 240245660—SFB1129 (project 8) (to BS). This work benefitted from the BioImage Light Microscopy Facility and Cell Analytics Facility of the University of Fribourg.

## Acknowledgments

We thank Flavian Thelen, César Nombela-Arrieta, Silvia F. Soriano and Fernanda Matos Coelho for support.

## Conflict of interest

The authors declare that the research was conducted in the absence of any commercial or financial relationships that could be construed as a potential conflict of interest.

## Publisher’s note

All claims expressed in this article are solely those of the authors and do not necessarily represent those of their affiliated organizations, or those of the publisher, the editors and the reviewers. Any product that may be evaluated in this article, or claim that may be made by its manufacturer, is not guaranteed or endorsed by the publisher.

## Supplementary material

Supplemental Movie 1 2PM image sequence showing WT (green) and p110δ^D910A/D910A^ (blue) B cell migration on FDC (white). Scale bar 40 µm; Time in min and s.

## References

[1] Förster R Mattis AE Kremmer E Wolf E Brem G Lipp M. A putative chemokine receptor, BLR1, directs b cell migration to defined lymphoid organs and specific anatomic compartments of the spleen. Cell (1996) 87:1037–47. doi: 10.1016/s0092-8674(00)81798-5 8978608

[2] Ansel KM Ngo VN Hyman PL Luther SA Förster R Sedgwick JD. A chemokine-driven positive feedback loop organizes lymphoid follicles. Nature (2000) 406:309–14. doi: 10.1038/35018581 10917533

[3] Park C Hwang I-Y Sinha RK Kamenyeva O Davis MD Kehrl JH. Lymph node b lymphocyte trafficking is constrained by anatomy and highly dependent upon chemoattractant desensitization. Blood (2012) 119:978–89. doi: 10.1182/blood-2011-06-364273 PMC3271721 22039261

[4] Coelho FM Natale D Soriano SF Hons M Swoger J Mayer J. Naive b-cell trafficking is shaped by local chemokine availability and LFA-1–independent stromal interactions. Blood (2013) 121:4101–9. doi: 10.1182/blood-2012-10-465336 23558016

[5] Fukui Y Hashimoto O Sanui T Oono T Koga H Abe M. Haematopoietic cell-specific CDM family protein DOCK2 is essential for lymphocyte migration. Nature (2001) 412:826–31. doi: 10.1038/35090591 11518968

[6] Nombela-Arrieta C Mempel TR Soriano SF Mazo I Wymann MP Hirsch E. A central role for DOCK2 during interstitial lymphocyte motility and sphingosine-1-phosphate–mediated egress. J Exp Med (2007) 204:497–510. doi: 10.1084/jem.20061780 17325199 PMC2137902

[7] Gadea G Blangy A. Dock-family exchange factors in cell migration and disease. Eur J Cell Biol (2014) 93:466–77. doi: 10.1016/j.ejcb.2014.06.003 25022758

[8] Kunimura K Uruno T Fukui Y. DOCK family proteins: key players in immune surveillance mechanisms. Int Immunol (2020) 32:5–15. doi: 10.1093/intimm/dxz067 31630188 PMC6949370

[9] Chen Y Chen Y Yin W Han H Miller H Li J. The regulation of DOCK family proteins on T and b cells. J Leukoc. Biol (2021) 109:383–94. doi: 10.1002/jlb.1mr0520-221rr 32542827

[10] Nombela-Arrieta C Lacalle RA Montoya MC Kunisaki Y Megıías D Marqués M. Differential requirements for DOCK2 and phosphoinositide-3-Kinase γ during T and b lymphocyte homing. Immunity (2004) 21:429–41. doi: 10.1016/j.immuni.2004.07.012 15357953

[11] Kunisaki Y Nishikimi A Tanaka Y Takii R Noda M Inayoshi A. DOCK2 is a rac activator that regulates motility and polarity during neutrophil chemotaxis. J Cell Biol (2006) 174:647–52. doi: 10.1083/jcb.200602142 PMC2064308 16943182

[12] Reif K Okkenhaug K Sasaki T Penninger JM Vanhaesebroeck B Cyster JG. Cutting edge: Differential roles for phosphoinositide 3-kinases, p110γ and p110δ, in lymphocyte chemotaxis and homing. J Immunol (2004) 173:2236–40. doi: 10.4049/jimmunol.173.4.2236 15294934

[13] Matheu MP Deane JA Parker I Fruman DA Cahalan MD. Class IA phosphoinositide 3-kinase modulates basal lymphocyte motility in the lymph node. J Immunol (2007) 179:2261–9. doi: 10.4049/jimmunol.179.4.2261 17675487

[14] Guillermet-Guibert J Bjorklof K Salpekar A Gonella C Ramadani F Bilancio A. The p110β isoform of phosphoinositide 3-kinase signals downstream of G protein-coupled receptors and is functionally redundant with p110γ. Proc Natl Acad Sci (2008) 105:8292–7. doi: 10.1073/pnas.0707761105 PMC2448830 18544649

[15] Nishikimi A Uruno T Duan X Cao Q Okamura Y Saitoh T. Blockade of inflammatory responses by a small-molecule inhibitor of the rac activator DOCK2. Chem Biol (2012) 19:488–97. doi: 10.1016/j.chembiol.2012.03.008 22520755

[16] Boscacci RT Pfeiffer F Gollmer K Sevilla AIC Martin AM Soriano SF. Comprehensive analysis of lymph node stroma-expressed ig superfamily members reveals redundant and nonredundant roles for ICAM-1, ICAM-2, and VCAM-1 in lymphocyte homing. Blood (2010) 116:915–25. doi: 10.1182/blood-2009-11-254334 PMC3324225 20395417

[17] Schymeinsky J Then C Sindrilaru A Gerstl R Jakus Z Tybulewicz VLJ. Syk-mediated translocation of PI3Kδ to the leading edge controls lamellipodium formation and migration of leukocytes. PloS One (2007) 2:e1132. doi: 10.1371/journal.pone.0001132 17987119 PMC2063580

[18] Cyster JG Allen CDC. B cell responses: Cell interaction dynamics and decisions. Cell (2019) 177:524–40. doi: 10.1016/j.cell.2019.03.016 PMC6538279 31002794

[19] Cosgrove J Novkovic M Albrecht S Pikor NB Zhou Z Onder L. B cell zone reticular cell microenvironments shape CXCL13 gradient formation. Nat Commun (2020) 11:3677. doi: 10.1038/s41467-020-17135-2 32699279 PMC7376062

[20] Okada T Ngo VN Ekland EH Foörster R Lipp M Littman DR. Chemokine requirements for b cell entry to lymph nodes and peyer’s patches. J Exp Med (2002) 196:65–75. doi: 10.1084/jem.20020201 12093871 PMC2194009

[21] Kanemitsu N Ebisuno Y Tanaka T Otani K Hayasaka H Kaisho T. CXCL13 is an arrest chemokine for b cells in high endothelial venules. Blood (2005) 106:2613–8. doi: 10.1182/blood-2005-01-0133 15972452

[22] Thelen F Wissmann S Ruef N Stein JV. The tec kinase itk integrates naïve T cell migration and *In vivo* homeostasis. Front Immunol (2021) 12:716405. doi: 10.3389/fimmu.2021.716405 34566971 PMC8458560

[23] Xu H Li X Liu D Li J Zhang X Chen X. Follicular T-helper cell recruitment governed by bystander b cells and ICOS-driven motility. Nature (2013) 496:523–7. doi: 10.1038/nature12058 23619696

[24] Henderson RB Grys K Vehlow A de Bettignies C Zachacz A Henley T. A novel rac-dependent checkpoint in b cell development controls entry into the splenic white pulp and cell survival. J Exp Med (2010) 207:837–53. doi: 10.1084/jem.20091489 PMC2856036 20308364

[25] de Gorter DJJ Beuling EA Kersseboom R Middendorp S van Gils JM Hendriks RW. Bruton’s tyrosine kinase and phospholipase Cγ2 mediate chemokine-controlled b cell migration and homing. Immunity (2007) 26:93–104. doi: 10.1016/j.immuni.2006.11.012 17239630

[26] Hoellenriegel J Meadows SA Sivina M Wierda WG Kantarjian H Keating MJ. The phosphoinositide 3′-kinase delta inhibitor, CAL-101, inhibits b-cell receptor signaling and chemokine networks in chronic lymphocytic leukemia. Blood (2011) 118:3603–12. doi: 10.1182/blood-2011-05-352492 PMC4916562 21803855

[27] de Rooij MFM Kuil A Geest CR Eldering E Chang BY Buggy JJ. The clinically active BTK inhibitor PCI-32765 targets b-cell receptor– and chemokine-controlled adhesion and migration in chronic lymphocytic leukemia. Blood (2012) 119:2590–4. doi: 10.1182/blood-2011-11-390989 22279054

[28] Sharma S Orlowski G Song W. Btk regulates b cell receptor-mediated antigen processing and presentation by controlling actin cytoskeleton dynamics in b cells. J Immunol (2009) 182:329–39. doi: 10.4049/jimmunol.182.1.329 PMC2855895 19109164

[29] Hons M Kopf A Hauschild R Leithner A Gaertner F Abe J. Chemokines and integrins independently tune actin flow and substrate friction during intranodal migration of T cells. Nat Immunol (2018) 19:606–16. doi: 10.1038/s41590-018-0109-z 29777221

[30] Jellusova J Rickert RC. The PI3K pathway in b cell metabolism. Crit Rev Biochem Mol Biol (2016) 51:359–78. doi: 10.1080/10409238.2016.1215288 PMC5139348 27494162

[31] Gollmer K Asperti-Boursin F Tanaka Y Okkenhaug K Vanhaesebroeck B Peterson JR. CCL21 mediates CD4+ T-cell costimulation *via* a DOCK2/Rac-dependent pathway. Blood (2009) 114:580–8. doi: 10.1182/blood-2009-01-200923 PMC2713469 19451552

[32] Hantschel O Rix U Schmidt U Bürckstümmer T Kneidinger M Schütze G. The btk tyrosine kinase is a major target of the bcr-abl inhibitor dasatinib. Proc Natl Acad Sci (2007) 104:13283–8. doi: 10.1073/pnas.0702654104 PMC1940229 17684099

[33] Wang ML Rule S Martin P Goy A Auer R Kahl BS. Targeting BTK with ibrutinib in relapsed or refractory mantle-cell lymphoma. N Engl J Med (2013) 369:507–16. doi: 10.1056/nejmoa1306220 PMC4513941 23782157

[34] Byrd JC Furman RR Coutre SE Flinn IW Burger JA Blum KA. Targeting BTK with ibrutinib in relapsed chronic lymphocytic leukemia. Np Engl J Med (2013) 369:32–42. doi: 10.1056/nejmoa1215637 PMC3772525 23782158

[35] Okkenhaug K Bilancio A Farjot G Priddle H Sancho S Peskett E. Impaired b and T cell antigen receptor signaling in p110δ PI 3-kinase mutant mice. Science (2002) 297:1031–4. doi: 10.1126/science.1073560 12130661

[36] Xu H Gonzalo JA Pierre YS Williams IR Kupper TS Cotran RS. Leukocytosis and resistance to septic shock in intercellular adhesion molecule 1-deficient mice. J Exp Med (1994) 180:95–109. doi: 10.1084/jem.180.1.95 7911822 PMC2191562

